# Acute Renal Infarction in a Patient Presenting With Abdominal Pain and Hypertensive Emergency

**DOI:** 10.7759/cureus.112429

**Published:** 2026-07-10

**Authors:** Syeda Salima Sultana, Mark Joseph Cerna Lopez, Nayeemul Islam, Winston Lee

**Affiliations:** 1 Internal Medicine, New York City (NYC) Health + Hospitals, South Brooklyn Health, Brooklyn, USA; 2 College of Medicine, Sir Salimullah Medical College, Dhaka, BGD; 3 Nephrology, New York City (NYC) Health + Hospitals, South Brooklyn Health, Brooklyn, USA

**Keywords:** abrupt malignant hypertension, acute renovascular ischemia, chronic end-organ, renal infarction, unexplained severe abdominal pain

## Abstract

Acute renal infarction is a rare but clinically important cause of abdominal pain and is frequently overlooked due to its nonspecific presentation. We report the case of a 46-year-old man who presented with progressive periumbilical abdominal pain and hypertensive emergency. Initial laboratory evaluation was nondiagnostic. Contrast-enhanced computed tomography demonstrated a wedge-shaped perfusion defect in the right kidney with segmental renal artery thrombosis, confirming acute renal infarction. Because his symptoms had been present for more than 72 hours, endovascular intervention was not pursued, and the patient was managed conservatively with systemic anticoagulation and aggressive blood pressure control. This case highlights the diagnostic challenges associated with renal infarction and emphasizes the importance of early imaging in patients presenting with abdominal pain and severe hypertension.

## Introduction

Acute renal infarction results from sudden interruption of renal arterial blood flow, most commonly due to cardioembolism or in situ thrombosis ​[1].​ Although potentially devastating, it remains markedly underdiagnosed. Reported incidence is extremely low with a range from 0.004% to 1.8%, depending on the population studied​ [2]. Clinical presentation is often nonspecific, with symptoms that overlap extensively with nephrolithiasis, pyelonephritis, and gastrointestinal disorders. Consequently, diagnostic delays are common and frequently exceed the narrow therapeutic window during which revascularization may preserve renal parenchyma ​[3]​. Severe hypertension represents an important but underrecognized diagnostic clue ​[4,5]​. Renal ischemia activates the renin-angiotensin-aldosterone system, leading to abrupt and sometimes malignant elevation in blood pressure. Recognition of this pathophysiologic relationship is critical for early diagnosis. This case illustrates the diagnostic pitfalls associated with acute renal infarction and underscores the need for heightened clinical suspicion in hypertensive emergencies. 

## Case presentation

A 46-year-old Hispanic male presented to the emergency department with a three-day history of progressive periumbilical abdominal pain radiating to the lower back and groin. The pain was constant, severe, and non-colicky, associated with constipation. Twenty-four hours before admission, he developed persistent nausea and repeated episodes of non-bilious, non-bloody vomiting. There were no urinary, gastrointestinal, cardiopulmonary, or traumatic symptoms to suggest alternative diagnoses. 

His medical history included poorly controlled hypertension and dyslipidemia. He admitted to nonadherence with antihypertensive therapy, having discontinued amlodipine approximately two months earlier. Home medications include 10 mg amlodipine once daily and atorvastatin 20 mg once daily. Social history was notable for a 15-year smoking history and alcohol consumption averaging 10 beers weekly. There was no personal or family history of thromboembolic disease.

On arrival, vital signs revealed severe hypertension, with a blood pressure of 219/132 mmHg. His heart rate was 83 beats per minute, respiratory rate 18 breaths per minute, temperature 36.6°C, and oxygen saturation was 96% on room air. Physical examination showed mild periumbilical tenderness without guarding, rebound, or costovertebral angle tenderness. Cardiopulmonary and neurologic examinations were unremarkable.

Laboratory evaluation demonstrated mild leukocytosis (12.38 × 10^9^/L). Serum creatinine was 0.8 mg/dL, and blood urea nitrogen was 11 mg/dL, indicating preserved renal function. Liver enzymes showed mild alanine aminotransferase (ALT) elevation. Serum lactate, lipase, and cardiac biomarkers were within normal limits. Urinalysis revealed no hematuria, proteinuria, or pyuria. Baseline laboratory investigations are summarized in Table 1. 

**Table 1:** Admission laboratory investigations. Admission laboratory evaluation demonstrating preserved renal function and absence of diagnostic abnormalities. Laboratory studies on admission revealed mild leukocytosis and preserved renal function, with no biochemical evidence of infection, pancreatitis, or tissue hypoperfusion. The absence of diagnostic laboratory abnormalities contributed to the delayed recognition of renal infarction. ALT: alanine aminotransferase; AST: aspartate aminotransferase.

Parameter	Result	Reference range
Hemoglobin	16.2 g/dL	13.5–17.5
White blood cell count	12.38 ×10⁹/L	4.0–11.0
Neutrophils	79.6%	40–70
Lymphocytes	11.1%	20–40
Platelets	318 ×10⁹/L	150–400
Serum creatinine	0.8 mg/dL	0.7–1.3
Blood urea nitrogen	11 mg/dL	7–20
Sodium	133 mmol/L	135–145
Potassium	4.0 mmol/L	3.5–5.0
Calcium	9.4 mg/dL	8.6–10.2
ALT	52 U/L	10–40
AST	34 U/L	10–40
Lipase	41 U/L	13–60
Serum lactate	1.4 mmol/L	0.5–2.0
Troponin (high sensitivity)	<6 ng/L	<14
Arterial pH	7.48	7.35–7.45
PaCO₂	33 mmHg	35–45
PaO₂	63 mmHg	75–100
Bicarbonate	25 mmol/L	22–26

Electrocardiography demonstrated sinus rhythm with voltage criteria for left ventricular hypertrophy. Chest radiography was unremarkable (Figure 1), excluding acute cardiopulmonary pathology and supporting a primary vascular etiology. Given the persistent abdominal pain and hypertensive emergency, contrast-enhanced computed tomography (CT) of the abdomen and pelvis was performed, revealing a sharply demarcated wedge-shaped region of hypoperfusion within the mid-pole of the right kidney (Figure 2), along with thrombosis of an inferior segmental branch of the right renal artery (Figure 3), confirming acute renal infarction.

**Figure 1:**
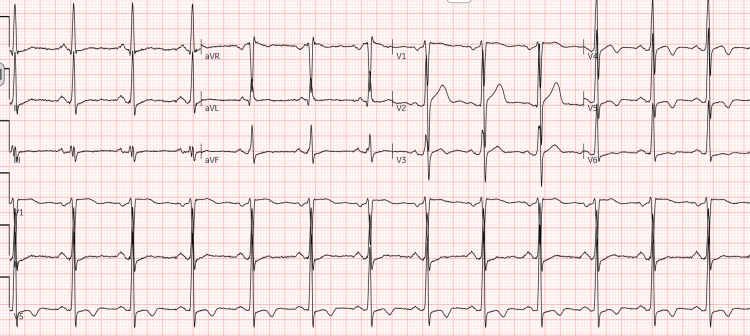
Twelve-lead ECG obtained on presentation showing normal sinus rhythm with voltage criteria for left ventricular hypertrophy (Sokolow–Lyon criteria) and nonspecific lateral T-wave inversion in leads V4–V6, consistent with chronic hypertensive heart disease and possible subendocardial ischemia.

**Figure 2:**
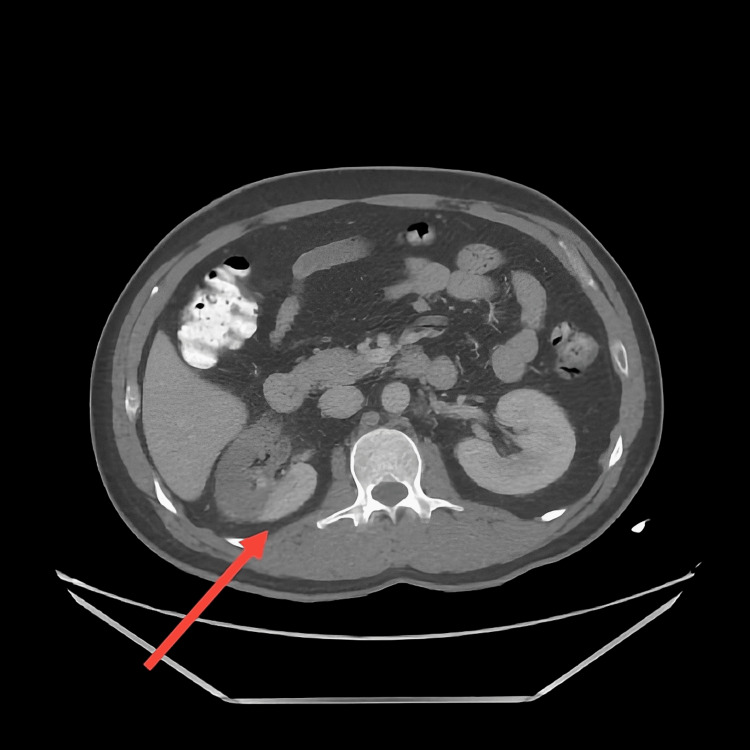
Axial contrast-enhanced CT image demonstrating a well-demarcated wedge-shaped hypodense perfusion defect in the mid-pole of the right kidney (arrow), consistent with acute renal infarction. CT: computed tomography.

**Figure 3:**
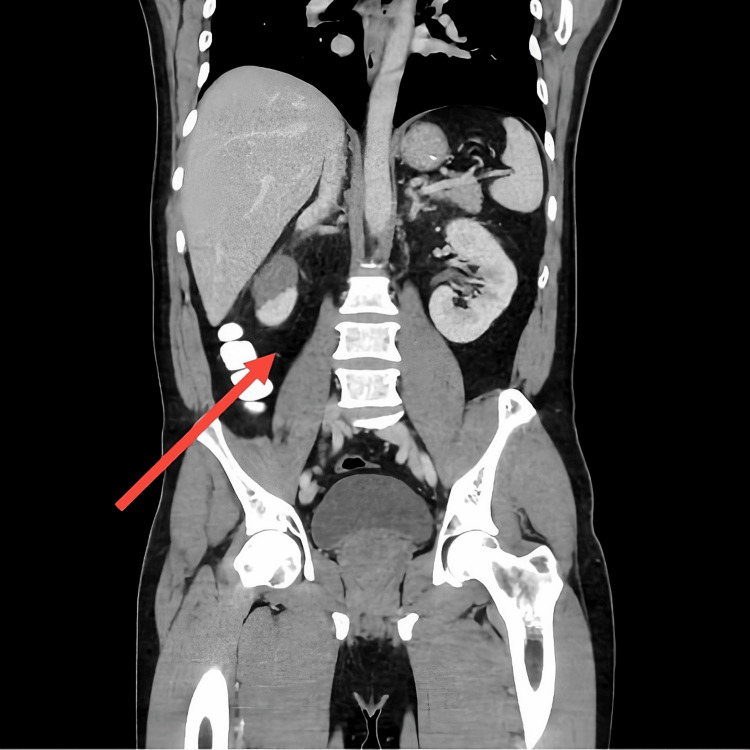
Contrast-enhanced coronal CT reconstruction demonstrating thrombus within the inferior segmental branch of the right renal artery supplying the ventral aspect of the kidney (arrow), corresponding to the perfusion defect seen on axial imaging. CT: computed tomography.

Transthoracic echocardiography demonstrated preserved biventricular systolic function, concentric left ventricular hypertrophy, and no intracardiac thrombus or valvular pathology. Continuous telemetry monitoring revealed no arrhythmias. Comprehensive hypercoagulable evaluation, including testing for inherited and acquired thrombophilia, was unremarkable. In the absence of cardioembolic sources, vascular dissection, or hypercoagulable states, the infarction was most consistent with in situ thrombosis secondary to uncontrolled hypertension and endothelial injury. 

Blood pressure was gradually controlled with intravenous hydralazine followed by nicardipine infusion. Systemic anticoagulation with intravenous unfractionated heparin was initiated. Interventional radiology and vascular surgery were consulted; however, revascularization was deferred due to symptom duration exceeding 72 hours, rendering renal salvage unlikely. The patient was transitioned to oral apixaban 5 mg twice daily, with a planned six-month course followed by long-term antiplatelet therapy. Antihypertensive therapy was optimized with losartan and amlodipine. 

Abdominal pain was resolved within 48 hours, and renal function remained stable throughout hospitalization. The patient was discharged on hospital day 3 with close outpatient follow-up.

## Discussion

Acute renal infarction is a frequently overlooked diagnosis because its clinical presentation is nonspecific and overlaps extensively with more common causes of abdominal and flank pain. Anchoring bias and premature diagnostic closure contribute significantly to delayed recognition, often beyond the therapeutic window for revascularization [4]. The principal etiologies of acute renal infarction and the recommended diagnostic approaches are summarized in Table 2 [6].

**Table 2:** Major causes of acute renal infarction and recommended diagnostic approach. CT: computed tomography.

Etiology	Typical clinical features	Key diagnostic tests	Implications for management
Cardioembolic (e.g., atrial fibrillation, mural thrombus, valvular disease)	Sudden onset flank or abdominal pain, history of arrhythmia, or cardiac disease	ECG, continuous cardiac monitoring, transthoracic ± transesophageal echocardiography	Systemic anticoagulation; consider revascularization if early presentation
In situ thrombosis (e.g., hypercoagulable state, endothelial injury)	Abdominal or flank pain, often with preserved renal function; may lack cardiac history	Hypercoagulable evaluation, CT angiography	Systemic anticoagulation; treat underlying cause
Renal artery dissection	Severe flank pain, sudden hypertension, possible trauma, or connective tissue disease	CT angiography or MR angiography	Endovascular or surgical repair depending on extent
Aortic pathology (e.g., mural thrombus, atheroembolism)	Abdominal pain, limb ischemia, systemic embolic phenomena	CT angiography of chest and abdomen	Anticoagulation ± vascular intervention
Idiopathic	Nonspecific abdominal pain, absence of identifiable cause	Diagnosis of exclusion	Anticoagulation and close surveillance

Acute renal infarction is most commonly associated with sudden onset of severe flank pain, nausea, vomiting, hematuria, elevated serum lactate dehydrogenase levels and, occasionally, acute kidney injury. However, unusual presentations are gaining recognition and can be mostly abdominal pain with minimal urinary changes or even high blood pressure without flank pain. These unusual presentations often resemble more typical gastrointestinal or hypertensive disorders, resulting in delayed diagnosis and reducing the time available for renal salvage.

In this case, preserved kidney function and the absence of flank pain contributed to diagnostic uncertainty. But it had a major clue because of the severity of the hypertension which occurred without explanation. There may be a bidirectional relationship between hypertension and renal infarction. Chronic hypertension can lead to vascular injury and thromboembolic events, and acute renal ischemia activates the RAAS, which can lead to sudden vasoconstriction and significant increases in blood pressure (the Goldblatt phenomenon) [7]. Recognizing this association may help in early diagnosis [8].

Contrast-enhanced CT remains the diagnostic modality of choice, allowing direct visualization of parenchymal perfusion deficits and vascular occlusion [8]. Endovascular revascularization may preserve renal tissue when performed within 24 hours of symptom onset, particularly in patients with main renal artery occlusion, bilateral involvement, or solitary kidneys. Beyond this period, irreversible ischemic injury predominates, limiting management to systemic anticoagulation and secondary prevention [9,10].

Systemic anticoagulation reduces thrombus propagation and the risk of recurrent embolism. Direct oral anticoagulants provide effective therapy with favorable safety profiles [8,9]. Strict blood pressure control and renin-angiotensin system blockade are essential to prevent recurrent ischemia and long-term renovascular complications [8,11].

## Conclusions

Acute renal infarction should be part of the differential diagnosis of acute abdominal pain, especially in cases of severe or unexplained hypertension. Sudden flank pain is the classic presentation; however, other symptoms may be presented such as abdominal pain with normal renal function, which may delay diagnosis. The relationship between hypertension and renal infarction is bidirectional: hypertension may predispose patients to renal infarction, while renal infarction may itself cause severe hypertension. Therefore, careful clinical evaluation is essential. Timely diagnosis and preservation of the kidney are important and require early contrast-enhanced imaging. In our patient, prompt initiation of anticoagulation and optimal blood pressure control resulted in early resolution of symptoms, intact renal function, and achievement of long-term oral anticoagulation without the need for revascularization.

## References

[1] Oh YK, Yang CW, Kim YL (2016). Clinical characteristics and outcomes of renal infarction. Am J Kidney Dis.

[2] Antopolsky M, Simanovsky N, Stalnikowicz R, Salameh S, Hiller N (2012). Renal infarction in the ED: 10-year experience and review of the literature. Am J Emerg Med.

[3] Faucon AL, Bobrie G, Jannot AS, Azarine A, Plouin PF, Azizi M, Amar L (2018). Cause of renal infarction: a retrospective analysis of 186 consecutive cases. J Hypertens.

[4] Bourgault M, Grimbert P, Verret C (2013). Acute renal infarction: a case series. Clin J Am Soc Nephrol.

[5] Paris B, Bobrie G, Rossignol P, Le Coz S, Chedid A, Plouin PF (2006). Blood pressure and renal outcomes in patients with kidney infarction and hypertension. J Hypertens.

[6] Domanovits H, Paulis M, Nikfardjam M, Meron G, Kürkciyan I, Bankier AA, Laggner AN (1999). Acute renal infarction. Clinical characteristics of 17 patients. Medicine (Baltimore).

[7] Van Epps HL (2005). Harry Goldblatt and the discovery of renin. J Exp Med.

[8] Saju JM, Diaz-Font J (2026). Renal infarction. StatPearls [Internet].

[9] Lengelé JP, Persu A, Noubouossie D (2018). Renal infarction management: towards an etiological approach. J Hypertens.

[10] Silverberg D, Menes T, Rimon U, Salomon O, Halak M (2016). Acute renal artery occlusion: presentation, treatment, and outcome. J Vasc Surg.

[11] Navar LG, Zou L, Von Thun A, Tarng Wang C, Imig JD, Mitchell KD (1998). Unraveling the mystery of Goldblatt hypertension. News Physiol Sci.

